# The effect of bio-banding on the anthropometric, physical fitness and functional movement characteristics of academy soccer players

**DOI:** 10.1371/journal.pone.0260136

**Published:** 2021-11-29

**Authors:** Calum MacMaster, Matt Portas, Guy Parkin, Sean Cumming, Chris Wilcox, Christopher Towlson

**Affiliations:** 1 School of Sport and Exercise Science, University of Birmingham, Birmingham, United Kingdom; 2 The English Football Association, St Georges Park, Burton Upton Trent, Staffordshire, United Kingdom; 3 School of Health and Science, Teesside University, Middlesbrough, Yorkshire, United Kingdom; 4 Pro Football Support, Huddersfield, Yorkshire, United Kingdom; 5 Department for Health, University of Bath, Bath, Somerset, United Kingdom; 6 Department of Sport, Health and Exercise Science, University of Hull, Yorkshire, United Kingdom; Universita degli Studi di Milano, ITALY

## Abstract

The study examined if maturity status bio-banding reduces within-group variance in anthropometric, physical fitness and functional movement characteristics of 319, under-14 and under-15 players from 19 UK professional soccer academies. Bio-banding reduced the within-bio-banded group variance for anthropometric values, when compared to an aggregated chronological banded group (chronological: 5.1–16.7%CV; bio-banded: 3.0–17.3%CV). Differences between these bio-banded groups ranged from *moderate* to *very large* (ES = 0.97 to 2.88). Physical performance variance (chronological: 4.8–24.9%CV; bio-banded: 3.8–26.5%CV) was also reduced with bio-banding compared to chronological aged grouping. However, not to the same extent as anthropometric values with only 68.3% of values reduced across banding methods compared to 92.6% for anthropometric data. Differences between the bio-banded groups physical qualities ranged from *trivial* to *very large* (ES = 0.00 to 3.00). The number of functional movement metrics and %CV reduced by bio-banding was lowest within the ‘circa-PHV’ groups (11.1–44.4%). The proportion of players achieving the threshold value score of ≥ 14 for the FMS™ was highest within the ‘post-PHV’ group (50.0–53.7%). The use of maturity status bio-banding can create more homogenous groups which may encourage greater competitive equity. However, findings here support a bio-banding maturity effect hypothesis, whereby maturity status bio-banding has a heightened effect on controlling for characteristics which have a stronger association to biological growth.

## Introduction

The unpredictable and highly individualised nature of the adolescent growth spurt which features accelerated phases of growth in stature (i.e., peak height velocity [PHV]) often leads to youth soccer players competing within the same chronological age grouping (i.e., under [U] 12–15 years), whilst exhibiting large variance in maturity-related anthropometric (typically stature) and some physical fitness development [1–3]. Despite coaches suggesting they place little value on temporary maturity-related characteristics [4], the relevance of within-age group variance of somatic, physical fitness and functional movement characteristics of players is of importance to talent selection and development practitioners, given that maturity-related enhancements in these characteristics can confound how players are subjectively evaluated [5] and increase the risk of sustaining a growth-related injury [6, 7]. For instance, maturity-related enhancements in anthropometric (i.e. stature) and some physical fitness (i.e. power, speed etc) characteristics can often contribute to the over-selection and representation of early maturing players [2] (and premature playing position allocation [1, 3]), who are the beneficiaries of early exposure to normative growth curves. In addition, maturity-related variance in growth can lead to some players within the under 11 to 15 age categories (typically between 9.7–10.7 years to 13.8–15.2 years [8, 9]) experiencing accelerated phases of growth in stature (+8.6 cm/year at 10.7 to 15.2 years) [8], while the associated musculature develops at a slower rate [10, 11]. This may result in between player differences in joint stiffness, bone density and functional movement capacity [7, 12] who are matched for age.

The combined effect of temporary anthropometrical and physical changes in player’s fitness and functional movement capacity may in part offer a plausible explanation for the “adolescent awkwardness,” phenomena [10, 11]. Whereby, aspects of the trunk and lower-limbs have increased in length and size, but the associated musculature and nervous system have yet to be sufficiently developed to meet the new demands imposed on them by the players enlarged skeletal frame, sometimes causing abnormal movement [10, 13]. This transient event has implications from a talent identification and athletic development perspective, with research highlighting the numerical grading of a player’s performance being negatively impacted by their growth spurt period [5]. Additionally, given that evidence exists to suggest that temporary, maturity-related development of anthropometric characteristics of adolescent soccer players may be associated to an increased risk of sustaining injury [6, 7], academy practitioners may wish to consider alternative methods for grouping players for both talent and athletic development purposes.

To control for the within-group, maturity-related variance in growth and physical fitness development, maturity status bio-banding has been implemented within-academy soccer programmes [14–18] as an alternative to chronologically ordered playing age groups. The typical intended use of maturity status bio-banding is to reduce the (likely) temporary anthropometric (e.g., superior stature) and physical (e.g., superior strength and power) fitness advantages afforded to early maturing players in comparison to their later maturing counterparts [19, 20], a concept which should be considered separate to relative age bias in soccer [21]. Permitting a playing or training environment which is suggested to comprise of a homogenous group of players who exhibit similar maturity-related anthropometric, physical fitness and functional movement characteristics. However, despite previous research studies examining the effect of bio-banding on key (technical, tactical, physical and psychological) match-play characteristics [14, 15, 18], accompanied by studies which have explored key stakeholders [22] and players [16, 17] perceptions of maturity status bio-banding. There remains no evidence to suggest that maturity status bio-banding is an effective method for reducing maturity associated variance in anthropometric, physical fitness and functional movement characteristics of young soccer players. In addition, although the limitations associated with estimating maturation status using original [23] and subsequent iterations [16–24] of the maturity-offset method are well discussed [11, 25–27], the maturity-offset measure remains a popular method within soccer academies for estimating the maturation status of academy soccer players [28] and has been used to bio-band players [14–18]. Therefore, the aim of this study was to examine if maturity status bio-banding using popular maturity offset measures (i.e. [23, 24, 29]) reduces the within-group variance of anthropometric, physical fitness and functional movement characteristics of academy soccer players. The study also aimed to explore the methodological effect on player group variance when using the Mirwald, Baxter-Jones [23], Fransen, Bush [24] and Moore, McKay [29] maturity off-set estimation equations to categorise players into distinct bio-banded groupings.

## Methods

Following ethical approval (University of Hull -1415038), a total of 319 U14 and U15 soccer players (age: 14.5 ± 0.8 years, stature 169.4 ± 8.7 cm, sitting height 86.5 ± 4.9 cm, body-mass 57.9 ± 9.7 kg) participated in the study. The players were participating in 19 (U14 (n = 18) and U15 (n = 14)) UK academy soccer programmes, governed by the English Elite Player Performance Plan (EPPP) (category 2: 1 club; category 3: 16 clubs; category 4: 2 clubs), and were required to perform a battery of three anthropometric, five physical fitness and seven functional movement and a postural assessments. Similar to previous work [2], as players were not required to complete any additional activities or interventions than what they would normally have performed during daily training activities, and that all activities completed by the players were dictated by the club as part of a pre-existing agreement between the club, players, and guardian/parents, written, informed consent was not considered necessary which was approved by the intuitional ethics board. However, each player, parent/guardian was informed that they were permitted to withdraw their data from the research at any point, without giving reason. All tests were conducted by trained staff who were Disclosure and Barring services approved. Each player was free from injury and cleared to train by medical staff and had been habituated to the individual components of the battery of field tests. The order of tests followed previously published recommendations [30] whereby players in a rested state completed the anthropometric (i.e., stature, sitting height, and body-mass) measures first. These were followed by postural and functional movement assessments, physical fitness tests (i.e., vertical counter-movement jump. T-Test, linear accelerations, and sprints) and finally a maximal fatigue test (i.e., Multi-Stage Fitness Test). Similar to previous work [3], the examined chronological playing age groups (U14 and U15) were aggregated to facilitate sufficiently powered contrasts between maturity status bio-banding groups. We consider this aggregation acceptable, given that bi-annual age grouping strategies have also been used previously to bio-band players [11].

### Anthropometric and maturity measures

Duplicate measures of player body-mass (seca© robusta 813, Chino, USA), sitting height and stature (seca© 217, Chino, U.S.A) were taken. As stated with previously published methods [3, 8, 14], if measures varied ≥ 0.4 kg or 0.4 cm, a third measure was taken, and median value recorded. Player leg-length was determined as stature minus sitting height. A combination of all the aforementioned anthropometric measures, alongside the players decimal age were used to estimate the age at onset of age of peak height velocity (APHV) using three cross validated algorithms [23, 24, 29]. Given the importance for accurate anthropometric measures [11, 31], the test-retest reliability for a sample of 45 academy soccer players (12–16 years) separated by 7-days has been established (stature: intraclass correlation [ICC] = 1.00 [1.00–1.00], typical error [TE] = 0.6 cm [0.5 cm– 0.7 cm]; sitting height: ICC = 0.97 [0.95–0.98]; TE = 0.9 cm [0.8 cm to 1.1 cm]; body-mass: ICC = 1.00 [1.00–1.00]; TE = 0.3 kg [0.3 kg– 0.4 kg] (See Towlson, Cobley [3]). To account for the increased level of inaccuracy in the estimation of players furthest away from age at PHV [32], a cut-off point of ± 2.0 years from age at PHV was set [32] which resulted in 319 players being selected for analysis.

### Physical fitness measures

Using previously outlined methods [2, 3, 8], player explosive power was measured using a vertical counter-movement jump (CMJ) (SmartJump©, Fusion Sport, Cooper Planes, Australia). Each player was instructed to perform 3 maximal vertical jumps with their hands placed on their hips throughout, interspaced by 3-minutes passive recovery. The test was preceded by players performing a warm-up jump at 50% and 75% of their self-determined maximum effort. If the range of the 3 highest jumps varied ≥ 0.4 cm, additional jumps were performed until the 3 best jumps satisfied the stated threshold and the mean height was taken. Timed (Brower Timing System, Salt Lake City, Utah, U.S.A.) agility was recorded using the T-Test [33]. Players were required to sprint forward 9.14 m, side shuffle (keeping a forward-facing position) left 4.75 m, return to the mid-point and replicate the actions for the opposite side of the course. Each players’ measure of agility was determined by the duration it took for them to navigate the T-shaped course. Prior to test completion, all players completed duplicate warm-up efforts at 75% of their self-determined maximum with both left and right direction efforts (total: n = 4), interspaced by 3-minutes of passive recovery. The fastest time for left and right-side efforts were recorded and the mean value was used to establish agility performance. Having performed a standardised warm-up (50%, 75% and 90% of self-determined maximum sprint effort), players performed three timed (Brower Timing System, Salt Lake City, Utah, U.S.A.) 10 and 20 m accelerations, interspaced by 3-minutes passive recovery. Timing gates were placed at 0, 10, and 20m and the mean time was recorded. Lastly, players endurance capacity was assessed using the multi-stage fitness test (MSFT) [34]. To ensure that the players achieved the correct timings for the initial stages (6–11 km/h), an experienced test administrator acted as a pacer. The running speed of the test increased by 1.0 km/h every 60 seconds, until test failure (volitional exhaustion, when the player was unable to keep pace with the audio signals to signify the completion of a required 20m shuttle). Total distance covered (m) was used for the MSFT outcome measure, given that maximal aerobic speed as determined by the MSFT is typically under-reported by ~ 3km/h [34].

### Functional movements and postural assessments

As per previously published methods [12], the present study followed guidelines for the functional movement screening (FMS™) [35]. Whereby, players were instructed to complete (in this order) an overhead squat, hurdle step, inline lunge, shoulder mobility, active straight leg raise, trunk stability push up and a rotary stability. After each test, players were awarded a score of 0 to 3 (3 being recognised as most competent) as per the guidance [35]. The composite test scores were then aggregated to produce a total FMS™ score (See Portas, Parkin [12] for full scoring details), with the maximum individual score of 3 for 7 tests being aggregated to provide a maximum score of 21. Cut off points of ≥ 2 for component tests and ≥ 14 were selected for proportional analysis (See Portas, Parkin [12] for full description). Postural assessment was conducted visually (See Tunnell [36] for full description).

### Statistical analysis

Examination of the anthropometric and physical scores for the aggregated chronological and maturity determined bio-banded groups was presented as mean values ± standard deviation (SD) values. Given the categorical nature of the FMS™ data, this was presented as the median value and interquartile range (IQR). The proportion of subjects achieving the FMS™ cut-off values and above were used to examine between chronological and bio-banded groups, presented as proportional percentage value. Differences in the level of variability for the anthropometric, physical and FMS™ measures between the aggregated chronological and the maturity determined bio-banded groups were calculated using coefficient of variance percentage (%CV) values. To highlight the magnitude of difference present between the bio-banded groups, effect sizes were calculated, and thresholds set at: Trivial <0.2, Small 0.2–0.6, Moderate 0.6–1.2, Large 1.2–2.0, Very Large > 2.0 [37]. The smallest worthwhile change (SWC) from the aggregated chronological values were also calculated to provide impact of the use of bio-banding compared to the current arrangement of youth players when comparing the measured values.

## Results

**Fig 1:**
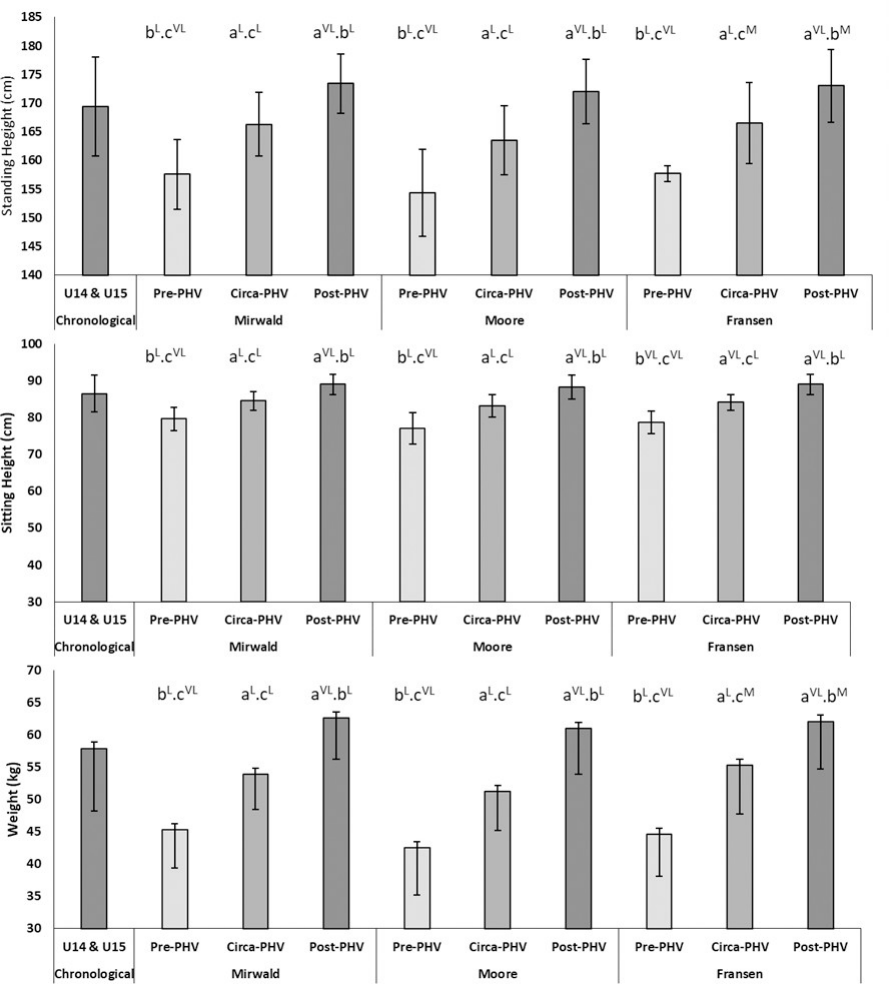
Comparison of mean values (+/ SD) between chronological (U14 & U15) and bio-banded maturity groups for anthropometric (stature, sitting height and body-mass) with effect sizes for between maturity status group (a—’pre-PHV’; b—’circa-PHV’; c–‘post-PHV) magnitude. Effect Sizes: T–Trivial, S–Small, M–Moderate, L–Large, VL–Very Large; Trivial < 0.2, Small 0.2–0.6, Moderate 0.6–1.2, Large 1.2–2.0, Very Large > 2.0 [37].

The mean (± S.D) anthropometric values for players within the aggregated chronological group and within maturity status determined bio-banded groups are presented within Fig 1. Anthropometric values increased according to advancing maturity status for all maturity offset estimations (Mirwald, Baxter-Jones [23], Fransen, Bush [24] and Moore, McKay [29]. The effect size differences between these groups ranged from *moderate* to *very large* (stature ES = 0.97 to 2.88; sitting height ES = 1.70 to 3.61; body-mass ES = 1.12 to 2.57) (Fig 1). The anthropometric values for all groups across the maturity status bio-banding methods exceeded the SWC. The %CV for the three anthropometric values was reduced (stature: CV = 3.0 to 4.9%; sitting height: CV = 2.7 to 5.6%; body-mass: CV = 10.1 to 17.3%) across all maturity bio-bands and methods of maturity estimation, except from the ‘pre-PHV’ group, when banded using the Moore, McKay [29] method (Table 1).

**Table 1:** Summary table of the coefficient of variance (CV%) for U13 and U14 academy soccer players anthropometric characteristics when categorised using an aggregated chronological age group or three different Mirwald, Baxter-Jones [23], Fransen, Bush [24] and Moore, McKay [29] bio-banding (pre-PHV, circa-PHV and post-PHV) methods.

Anthropometric variable	Chronological	Bio-banded (Mirwald et al. 2002)				Bio-banded (Moore et al. 2015)				Bio-banded (Fransen et al. 2018)			
	U13 & U14 CV%	Pre-PHV CV%	Circa-PHV CV%	Post-PHV CV%	Mean CV% (SD)	Pre-PHV CV%	Circa-PHV CV%	Post-PHV CV%	Mean CV% (SD)	Pre-PHV CV%	Circa-PHV CV%	Post-PHV CV%	Mean CV% (SD)
	*n* = 319	*n* = 40	*n* = 119	*n* = 116		*n* = 15	*n* = 113	*n* = 137		*n* = 35	*n* = 115	*n* = 134	
**Stature (cm)**	5.1	3.9	3.4	3.0	3.4 (± 0.5)	4.9	3.7	3.3	4.0 (± 0.8)	4.7	4.3	3.6	4.2 (± 0.6)
**Sitting Height (cm)**	5.6	3.9	2.9	3.1	3.3 (± 0.5)	5.6	3.6	3.7	4.3 (± 1.1)	3.8	2.7	3.0	3.2 (± 0.6)
**Body-mass (kg)**	16.7	13.0	10.2	10.1	11.1 (± 1.6)	17.3	11.8	11.7	13.6 (± 3.2)	14.6	14.0	12.0	13.5 (± 1.4)
**No of metrics showing CV% decrease**		**3 of 3**	**3 of 3**	**3 of 3**	**3 of 3**	**1 of 3**	**3 of 3**	**3 of 3**	**3 of 3**	**3 of 3**	**3 of 3**	**3 of 3**	**3 of 3**
**% of metrics showing CV% decrease**		**100%**	**100%**	**100%**	**100%**	**33%**	**100%**	**100%**	**100%**	**100%**	**100%**	**100%**	**100%**

CV%—Percentage coefficient of variance; SD—Standard deviation; PHV -Peak height velocity.

Table 2 shows the physical fitness characteristics of players improved towards and beyond PHV, with the ‘post-PHV’ players recording superior physical performance measures. The magnitude of these physical fitness differences between the maturity status groups ranges from *trivial* to *very large* (ES = 0.00 to 3.00). The largest of these differences are present within tests of speed including the 10m, 20m and agility run (10m: 1.00 to 2.00; 20m: 0.50 to 3.00; agility run: 0.33 to 1.40) (*small* to *very large*). The number of physical fitness metrics decreasing in %CV were less compared to the anthropometrical values (anthropometric: 25 of 27 = 92.6%; physical: 43 of 63 = 68.3%). ‘Circa-PHV’ players displayed an inferior number of metrics showing a reduction in %CV when compared to the aggregated chronological group, which was evidenced by the range being 11.1% - 44.4% across maturity status bio-banding methods. In addition, they also showed a lower number of metrics presenting a reduced %CV when compared to ‘pre-PHV’ (44.4%– 66.9%) and the ‘post-PHV’ (44.4%– 66.7%) groups across the three estimation methods. There were no values in the bio-banded groups which exceeded the SWC for the CMJ and total distance MSFT for all maturity status bio-banding methods (Table 3). For measures of speed, values exceeding the SWC are seen within the ‘pre-PHV’ and ‘post-PHV’ groupings for 10m and all maturity status bio-bandings with the exception of the ‘post-PHV’ group under the Moore, McKay [29] protocol for the 20m test (Table 3).

**Table 2:** Summary table of the mean values and between maturity grouping effect sizes for U13 and U14 academy soccer players physical fitness characteristics when categorised using an aggregated chronological age group or three different Mirwald, Baxter-Jones [23], Fransen, Bush [24] and Moore, McKay [29] bio-banding (pre-PHV, circa-PHV and post-PHV) methods.

Physical fitness variable	Chronological		Bio-banded (Mirwald et al. 2002)			Bio-banded (Moore et al. 2015)			Bio-banded (Fransen et al. 2018)		
	U13 & U14 (Mean ± S.D)	Smallest Worthwhile Change	Pre-PHV (Mean ± S.D)	Circa-PHV (Mean ± S.D)	Post-PHV (Mean ± S.D)	Pre-PHV (Mean ± S.D)	Circa-PHV (Mean ± S.D)	Post-PHV (Mean ± S.D)	Pre-PHV (Mean ± S.D)	Circa-PHV (Mean ± S.D)	Post-PHV (Mean ± S.D)
	*n* = 319		*n* = 40	*n* = 119	*n* = 116	*n* = 15	*n* = 113	*n* = 137	*n* = 35	*n* = 115	*n* = 134
**CMJ (cm)**	28.8 (± 7.2)	1.4	28.0 (± 6.1)	28.4 (± 7.3)	29.5 (± 7.6)	28.6 (± 5.8)	28.6 (± 6.7)	29.5 (± 7.7)	28.8 (± 5.6)	28.3 (± 7.5)	29.1 (± 7.4)
			Circa T, Post S	Pre T, Post T	Pre S, Circa T	Circa T, Post T	Circa T, Post T	Circa T, Post T	Circa T, Post T	Pre T, Post T	Pre T, Circa T
**Total Distance MSFT (m)**	1956.6 (± 297.3)	59.5	1875.9 (± 258.7)	1914.1 (± 298.4)	2025.6 (± 306.2)	1826.7 (± 251.5)	1900.0 (± 303.6)	1974.4 (± 292.7)	1872.9 (± 257.9)	1922.5 (± 309.1)	2010.6 (± 292.2)
			Circa T, Post S	Pre T, Post S	Pre S, Circa S	Circa S, Post S	Circa S, Post S	Circa S, Post S	Circa T, Post S	Pre T, Post S	Pre S, Circa S
**Maximal Aerobic Speed (m.sec-1)**	13.6 (± 0.6)	0.1	13.5 (± 0.6)	13.5 (± 0.7)	13.8 (± 0.7)	13.4 (± 0.6)	13.5 (± 0.7)	13.7 (± 0.6)	13.5 (± 0.6)	13.6 (± 0.7)	13.8 (± 0.6)
			Circa T, Post S	Pre T, Post S	Pre S, Circa S	Circa T, Post S	Pre T, Post S	Pre S, Circa S	Circa T, Post S	Pre T, Post S	Pre S, Circa S
**Velocity at Exhaustion (m.sec** ^**-1**^ **)**	13.4 (± 0.7)	0.1	13.3 (± 0.6)	13.3 (± 0.7)	13.6 (± 0.7)	13.2 (± 0.6)	13.3 (± 0.7)	13.5 (± 0.6)	13.3 (± 0.6)	13.4 (± 0.7)	13.6 (± 0.6)
			Circa T, Post S	Pre T, Post S	Pre S, Circa S	Circa T, Post S	Pre T, Post S	Pre S, Circa S	Circa T, Post S	Pre T, Post S	Pre S, Circa S
**10m (sec)**	1.7 (± 0.1)	0.0	1.8 (± 0.1)	1.7 (± 0.1)	1.6 (± 0.1)	1.8 (± 0.1)	1.7 (± 0.1)	1.6 (± 0.1)	1.8 (± 0.1)	1.7 (± 0.1)	1.6 (± 0.1)
			Circa M, Post L	Pre M, Post M	Pre L, Circa M	Circa M, Post L	Pre M, Post M	Pre L, Circa M	Circa M, Post L	Pre M, Post M	Pre L, Circa M
**20m (sec)**	3.0 (± 0.2)	0.0	3.2 (± 0.1)	3.1 (± 0.2)	2.9 (± 0.1)	3.2 (± 0.1)	3.1 (± 0.2)	3.0 (± 0.2)	3.2 (± 0.2)	3.1 (± 0.2)	2.9 (± 0.1)
			Circa M, Post VL	Pre M, Post L	Pre VL, Circa L	Circa M, Post L	Pre M, Post S	Pre L, Circa S	Circa S, Post L	Pre S, Post L	Pre L, Circa L
**Agility Mean Time (sec)**	10.0 (± 0.6)	0.1	10.3 (± 0.5)	10.1 (± 0.6)	9.9 (± 0.6)	10.6 (± 0.4)	10.2 (± 0.6)	9.9 (± 0.6)	10.4 (± 0.6)	10.1 (± 0.6)	9.9 (± 0.6)
			Circa S, Post M	Pre S, Post S	Pre M, Circa S	Circa M, Post L	Pre M, Post S	Pre L, Circa S	Circa S, Post M	Pre S, Post S	Pre M, Circa M

SD—Standard deviation; PHV -Peak height velocity; CMJ–Counter-movement jump; MSFT–Multi-stage Fitness Test; Effect Sizes: T—Trivial, S—Small, M—Moderate, L—Large, VL—Very Large; Trivial < 0.2, Small 0.2 0.6, Moderate 0.6–1.2, Large 1.2–2.0, Very Large >2.0 [37]; Note: lower values for tests of speed and agility represent better performance.

**Table 3:** Summary table of the coefficient of variance (CV%) for U13 and U14 academy soccer players physical fitness characteristics when categorised using an aggregated chronological age group or three different Mirwald, Baxter-Jones [23], Fransen, Bush [24] and Moore, McKay [29] bio-banding (pre-PHV, circa-PHV and post-PHV) methods.

Physical fitness variable	Chronological	Bio-banded (Mirwald et al. 2002)				Bio-banded (Moore et al. 2015)				Bio-banded (Fransen et al. 2018)			
	U13 & U14 CV%	Pre-PHV CV%	Circa-PHV CV%	Post-PHV CV%	Mean CV% (SD)	Pre-PHV CV%	Circa-PHV CV%	Post-PHV CV%	Mean CV% (SD)	Pre-PHV CV%	Circa-PHV CV%	Post-PHV CV%	Mean CV% (SD)
	*n* = 319	*n* = 40	*n* = 119	*n* = 116		*n* = 15	*n* = 113	*n* = 137		*n* = 35	*n* = 115	*n* = 134	
**CMJ (cm)**	24.9	21.8	25.8	25.7	24.4 (± 2.3)	20.4	23.4	26.0	23.3 (± 2.8)	20.3	26.5	25.4	24.1 (± 3.3)
**Total Distance MSFT (m)**	15.2	13.8	15.6	15.1	14.8 (± 0.9)	13.8	16.1	14.8	14.9 (± 1.2)	13.8	16.1	14.5	14.8 (± 1.2)
**Maximal Aerobic Speed (m.sec** ^**-1**^ **)**	4.8	4.3	4.8	4.8	4.6 (± 0.3)	4.2	5.0	4.6	4.6 (± 0.4)	4.4	5.0	4.6	4.7 (± 0.3)
**Velocity at Exhaustion (m.sec** ^**-1**^ **)**	4.8	4.3	4.9	4.9	4.3 (± 0.3)	4.4	5.2	4.6	4.7 (± 0.4)	4.3	5.0	4.7	4.7 (± 0.4)
**10m (sec)**	6.8	5.6	7.0	4.7	5.8 (± 1.2)	5.5	6.9	5.0	5.8 (± 1.0)	5.8	6.5	4.7	5.7 (± 0.9)
**20m (sec)**	6.2	4.3	6.3	4.7	5.1 (± 1.1)	4.6	5.8	5.2	5.2 (± 0.6)	4.8	6.0	4.6	5.1 (± 0.8)
**Agility Mean Time (sec)**	6.1	5.2	5.6	6.2	5.7 (± 0.5)	3.8	5.6	5.9	5.1 (± 1.1)	5.3	5.7	6.0	5.7 (± 0.4)
**No of metrics showing CV% decrease**		**7 of 7**	**1 of 7**	**3 of 7**	**7 of 7**	**7 of 7**	**3 of 7**	**6 of 7**	**7 of 7**	**7 of 7**	**3 of 7**	**6 of 7**	**7 of 7**
**% of metrics showing CV% decrease**		**100.0%**	**14.3%**	**42.9%**	**100.0%**	**100.0%**	**42.9%**	**85.7%**	**100.0%**	**100.0%**	**42.9%**	**85.7%**	**100.0%**

CV%—Percentage coefficient of variance; SD—Standard deviation; PHV -Peak height velocity; CMJ–Counter-movement jump; MSFT–Multi-stage Fitness Test.

Table 4 presents the number of metrics displaying a decrease in %CV for the maturity status bio-banded group FMS™ values in comparison to the chronological group which was lowest for the ‘circa-PHV’ (11.1–44.4%) group for the three bio-banding methods. Indicating the highest levels of variance within this group in comparison to the other bio-banded cohorts (‘pre-PHV’: 44.4–66.9%; ‘post-PHV’: 44.4–66.7%). The median (IQR) values for the FMS™ and posture tests across the chronological and bio-banded groups.

**Table 4:** Summary table of the coefficient of variance (CV%) for U13 and U14 academy soccer players functional movement characteristics when categorised using an aggregated chronological age group or three different Mirwald, Baxter-Jones [23], Fransen, Bush [24] and Moore, McKay [29] bio-banding (pre-PHV, circa-PHV and post-PHV) methods.

Functional movement variable	Chronological	Bio-banded (Mirwald et al. 2002)				Bio-banded (Moore et al. 2015)				Bio-banded (Fransen et al. 2018)			
	U13 & U14 CV%	Pre-PHV CV%	Circa-PHV CV%	Post-PHV CV%	Mean CV% (SD)	Pre-PHV CV%	Circa-PHV CV%	Post-PHV CV%	Mean CV% (SD)	Pre-PHV CV%	Circa-PHV CV%	Post-PHV CV%	Mean CV% (SD)
	*n* = 319	*n* = 40	*n* = 119	*n* = 116		*n* = 15	*n* = 113	*n* = 137		*n* = 35	*n* = 115	*n* = 134	
**Deep Squat (AU)**	26.0	26.0	28.8	25.1	26.6 (± 1.9)	21.5	29.0	26.4	25.6 (± 3.8)	26.5	26.5	27.2	26.7 (± 0.4)
**Hurdle Step (AU)**	34.5	33.9	35.9	33.9	34.6 (± 1.8)	35.1	37.5	32.0	34.9 (± 2.8)	32.6	35.7	34.9	34.4 (± 1.6)
**In-Line Lunge (AU)**	31.8	31.5	37.7	26.2	31.8 (± 5.8)	16.6	38.5	29.1	28.1 (± 11.0)	30.2	36.4	28.4	31.7 (± 4.2)
**Active Straight Leg Raise (AU)**	33.5	32.0	32.7	35.4	33.4 (± 1.8)	23.6	33.7	35.3	30.9 (± 6.3)	29.9	33.1	36.1	33.0 (± 3.1)
**Trunk Stability Push-Up (AU)**	37.9	40.2	38.6	32.4	37.1 (± 4.1)	36.9	40.7	33.6	37.1 (± 3.6)	41.2	38.3	35.5	38.3 (± 2.9)
**Rotary Stability (AU)**	20.1	20.7	21.5	18.4	20.2 (± 1.6)	23.4	22.2	19.0	21.5 (± 2.3)	25.7	19.2	19.8	21.6 (± 3.6)
**Shoulder Mobility (AU)**	27.1	21.7	27.0	30.2	26.3 (± 4.3)	25.0	24.1	29.5	26.2 (± 2.9)	20.8	25.0	30.2	25.3 (± 4.7)
**Posture (AU)**	24.4	28.8	23.8	23.0	25.2 (± 3.1)	39.7	25.5	22.5	29.2 (± 9.2)	28.8	25.3	22.8	25.6 (± 3.0)
**FMS (AU)**	14.1	15.8	14.1	14.7	14.9 (± 0.9)	13.3	14.8	14.9	14.3 (± 0.9)	16.4	14.0	14.6	15.0 (± 1.2)
**No of metrics showing CV% decrease**		4 of 9	3 of 9	6 of 9	3 of 9	6 of 9	1 of 9	5 of 9	5 of 9	4 of 9	4 of 9	4 of 9	4 of 9
**% of metrics showing CV% decrease**		44.4	33.3	66.7	33.9	66.9%	11.1%	55.6%	55.6%	44.4%	44.4%	44.4%	44.4%

CV%—Percentage coefficient of variance; SD—Standard deviation; PHV -Peak height velocity; AU—Arbitrary units; FMS–Functional Movement Screen.

**Fig 2:**
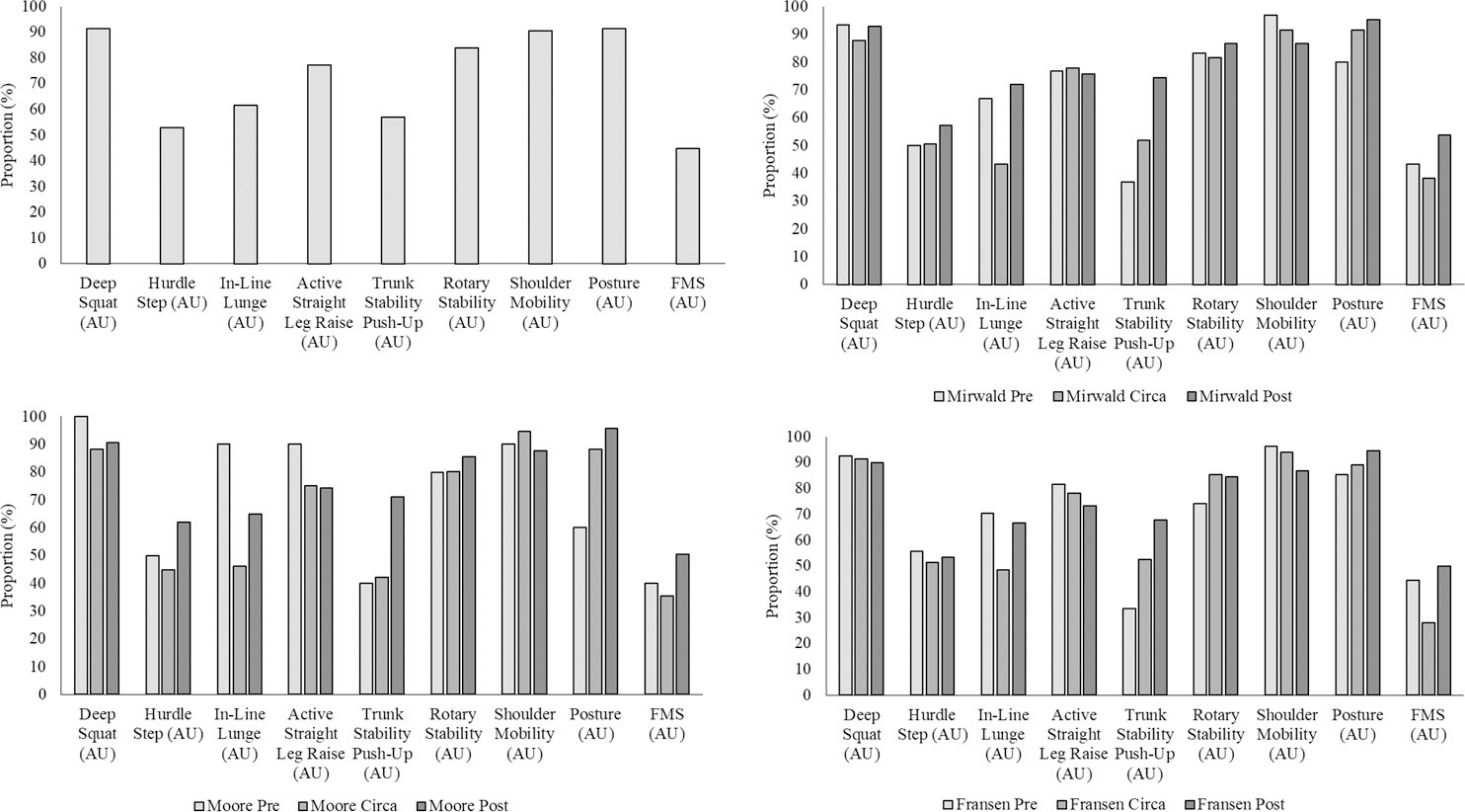
Comparison of proportion of players within each grouping (a—chronological or maturity status; b–Mirwald, Baxter-Jones. [23] c–Moore, McKay. [29]; d–Fransen, Bush [24]) bio-banding ‘pre = PHV’ (pre), ‘circa-PHV’ (circa) and ‘post-PHV’ (post)) achieving the threshold value of ≥2 (≥14 for composite FMS) for all measures of functional movement and postural assessment.

Fig 2 presents the proportion of players equal to or above the threshold value of 14 (out of 21) for the composite FMS™ value was lowest within the ‘circa-PHV’ (28.0% - 38.3%) groups across all three methods of maturity status estimation compared to ‘pre-PHV’ (40.0% - 44.4%) and ‘post-PHV’ (50.0% - 53.7%). Composites of the FMS™, the trunk stability push-up and a measure of posture assessment, highlighted a trend of increasing competency as maturity status also increased (trunk stability push-up: ‘pre-PHV: 33.3%– 40.0%; ‘circa-PHV’: 42.1% - 52.4%; ‘post-PHV’: 67.8% - 74.4; posture: ‘pre-PHV’: 60.0% - 85.2%; ‘circa-PHV’: 89.0% - 91.4%; ‘post-PHV’: 94.4% - 95.8%). A converse trend was seen for the shoulder mobility assessment, albeit not to the same level of proportional difference (‘pre-PHV’: 90.0% - 96.7%; ‘circa-PHV’: 94.7% - 91.4%; ‘post-PHV’: 87.6% - 86.6%).

## Discussion

The aim of the present study was to examine if popular [28] maturity offset measures (i.e. Mirwald, Baxter-Jones [23], Fransen, Bush [24] and Moore, McKay [29]) used to bio-band academy soccer players [14, 18] reduces the within-group variance of anthropometric, physical fitness and functional movement characteristics of ‘pre-PHV’, ‘circa-PHV’ and ‘post-PHV’ academy soccer players, compared to traditional aggregated (i.e., U14 and U15 soccer players) chronological groupings. Findings here are three-fold: 1) Maturity status bio-banding reduced the within-group variance for anthropometric variables, across all maturity status bio-banded groups (‘pre-PHV’, ‘circa-PHV’ and ‘post-PHV’), for each maturity status bio-banding method, with the exception of the ‘pre-PHV’ players banded by the Moore, McKay [29] equation; 2) Overall, maturity bio-banding reduced the within-group variance of academy soccer players physical fitness characteristics. Although the magnitude of change was more varied, with the number of physical metrics that displayed a reduction in %CV compared to the chronological group were less within the ‘circa-PHV’ groups across the three banding methods in comparison to the ‘pre-PHV’ and ‘post-PHV’ groupings; 3) In general, maturity status bio-banding had a limited effect on reducing the within-group variance of FMS™ characteristics, with only a small proportion of metrics reducing in comparison to chronological grouping.

Typically, the intended purpose of maturity status bio-banding is to reallocate players into groups characterised by homogenous maturity-related anthropometric and physical profiles [19, 20]. However, evidence to specifically examining the efficacy of this is absent. The present study has shown that maturity status bio-banding is an effective strategy for creating discrete homogenised groups of players, with each independent group characterised as possessing clear between-group anthropometric differences (i.e., exceeded the SWC thresholds). Both the ‘pre-PHV’ and ‘circa-PHV’ bio-banded groups presented a mean value lower than the chronological aged group for stature, sitting height and body-mass, with the ‘post-PHV’ individuals displaying larger values. This formation of anthropometrically different groups of players further supports the concept of maturity status bio-banding for producing more equitable playing and training environments for the adolescent soccer players.

The reduction of within-group variance in anthropometrica measures were likely due to the biological relationship which exists between the tempo of stature development and maturation [8, 9, 38]. Whereby, those players who are beneficiaries of earlier exposure to PHV are categorised with similar maturing players to create a homogeneous group of players, which can be repeated across maturity status spectrum (i.e., ‘pre’, ‘circa’ and ‘post-PHV’) as shown in the present study. Only one recorded exception was found within ‘pre-PHV’ players during the Moore, McKay [29] condition, where the variance in player body-mass increased (CV = 17.3%). However, this group also presented the smallest sample size (n = 15), perhaps rendering this finding as a statistical artifact due to the issues related to the use of small sample size [39]. Providing support to this argument is the finding that the ‘pre-PHV’ group within Moore, McKay [29] grouping also presented the largest SD values when compared to the same maturity status banding using the other maturity status bio-banding methods, for anthropometric measures.

Despite influencing anthropometric characteristics, the effect of maturity status bio-banding on physical fitness characteristics deemed important during talent identification [4] were reduced. This was evidenced by the reduced overall decrease in the number of measures showing a reduction of %CV (anthropometric: 92.6%; physical: 68.3%). In addition, only aerobic fitness metrics and measures of speed testing (10m, 20m, agility T-test) exceeded the SWC value. Therefore, it is possible that these metrics may have displayed a bio-banding effect due the fact that locomotor tests involve the displacement of body-mass, which is associated to advancing maturation as peak weight velocity (⁓7.5 kg/year) typically onsets at 11.9–16.1 years of age or −1.6 to +4.0 years to PHV [8]. This perhaps offers support for a bio-banding maturity effect hypothesis. Suggesting that maturity status bio-banding has a heightened effect on controlling for characteristics which have a stronger association to biological growth pathways and a diminishing effect the further removed the measured characteristic is from biological growth pathways. This hypothesis can in part be explained using previous literature which has assessed the relationship between maturation (of which maturity status bio-banding is based upon) with anthropometric and physical characteristics [6]. Consistent with the findings in this study, stature and body-mass measures were clearly affected by maturity status bio-banding, along with measures of speed. However, other physical metrics, such as lower limb power, were less obviously impacted by maturation, albeit they displayed a relatively consistent linear developmental rate [6]. That said, maturity status bio-banding still shows the creation of physically similar groupings in comparison to the chronological arrangement. The influence exerted by maturation on speed, power and aerobic fitness development have previously been highlighted when examining the influence of advanced stage of maturation has upon success within a talent identification environment [2, 40]. Players of advanced maturation (i.e., ‘post-PHV) are often afforded temporary maturity-related enhancements in physical fitness characteristics (e.g., faster speed and greater aerobic fitness levels), due to hormonal responses as a result of earlier pubertal onset which can influence the improvement of some physical qualities, such as strength and sprint ability [11, 41]. Therefore, suggesting that the implementation of maturity status bio-banding is a positive step in creating a more equitable playing environment for players by reducing the variance of physical qualities between players within a single banded group compared to the chronological age categorised talent development system.

Although evidence here suggests that maturity status bio-banding likely reduces within-group variance in both anthropometric and physical fitness qualities, it is currently unknown if maturity status bio-banding is an effective strategy to reduce maturity-related injuries associated to temporary biomechanical deficiencies at the onset of PHV [7, 42]. Functional movement assessments have highlighted the possible presence of an ‘adolescent awkwardness’ phase, defined as “delays or regression in sensorimotor function” during the period of the rapid growth spurt at PHV [10] which has highlighted the influence of maturational development on functional movement performance. With the application of maturity status bio-banding, the within-group variance was reduced for some functional movement measures within the ‘pre-PHV’ and ‘post-PHV’ groups, although no clear trend or specific functional movement could be determined. The maturity status bio-banded players categorised as ‘circa-PHV’ presented a greater level of variance for the majority of metrics in comparison to both the chronological and other maturity status bio-banded groups. As the ‘circa-PHV’ group spans ± 1.0 YPHV, there is potential for large variance to exist within the players with respect to their development in relation to PHV. This banding likely incorporates the adolescent growth spurt and the likely phase of accelerated growth in stature [8] in conjunction with a transient and short lived decline in motor performance, due to muscular and neural development being slower to ‘mature’ [10, 13]. As such, players within this grouping may have not yet experienced, are currently within or have recovered from this period of reduced motor control, contributing to the higher levels of variance seen.

Proportional analysis found the ‘post-PHV’ group to present the greatest level of functional movement competency (≥ 2 of 3 for component tests; ≥ 14 of 21 for cumulative FMS™ score). This finding also agrees with previous research [12, 13, 43] which found the most developed players (‘post-PHV’) likely record the highest overall FMS™ scores, displaying a superior functional movement ability than ‘pre-PHV’ individuals. The present results also highlight the ‘circa-PHV’ group recording the lowest FMS™ scores within maturity status bio-banded youth soccer players. An aggregated score of under 14 has been linked with an increased risk of serious injury [44]. As evidenced in the present study, the increased variance in movement skills and the reduction of movement quality emphasise the importance of appropriate loading of physical work for players around age at PHV onset due to the potential increased injury risk [7] through a short transitional decline in functional movement competency. Additionally, upper limb mobility as assessed by the shoulder mobility test, appears to be linked to maturational status, with the proportion of ‘post-PHV’ players scoring ≥2 (86.6–87.6%) being less than the ‘pre-PHV’ group (96.7–90.0%). As previously suggested [12], the reduction in upper limb mobility may be linked to the introduction of upper trunk resistance exercises (e.g. bench press), likely to become more prevalent in older athletes. However, it is also likely that these exercises in addition to the greater muscular development and neural control of the trunk have resulted a greater proportion of ‘post-PHV’ players achieving a ‘satisfactory’ (≥ 2) score for the ‘trunk stability push-up’ and posture assessments [45]. Therefore, it would be reasonable to include upper limb mobility work alongside upper trunk resistance exercises, especially in mature individuals.

The maturity offset measures used in the present study have been shown to possess reduced prediction accuracy for individuals furthest away from their APHV [24, 32]. To account for this, players deemed as ±2 YPHV were excluded from the study. Attempts have been made to reduce the prediction error from early maturity offset equations [23], resulting in more accurate methods [24]. However, some prediction error was still anticipated, and caution is always advised when interpreting both maturity offset and percentage of adult height attainment based estimations [11, 27]. A particular concern attributed to the use of maturity offset measures is the over- and under- estimation of age at PHV in late and early maturing males [24, 32]. As such, these individuals perhaps represent a “regressed to the mean” and consequently are likely to be assigned to equivalent maturity bands [32]. This limitation may, to some extent, help explain the limited success of the strategy to reduce maturity associated variance in both size and function. As such, caution when using this method to bio-band youth soccer players for training or match purposes should be taken by practitioners. That said recent research has suggested the use of percentage of adult height attained may provide a more reliable estimation of PHV [26]. However, at the time of data collection, this method was not feasible given the limited access to adequate numbers of parental heights required. Differences between the bio-bandings used were minimal, with all anthropometric values reporting similar trends. Findings here suggest little methodological effect on anthropometric, physical fitness and FMS™ values when bio-banded. Through examination of the mean response, all methods reduce variance in anthropometric and physical fitness values to a similar degree, whilst the Moore et al. [29] equation appears most effective at reducing FMS™ the most, however, this finding is currently limited to this study.

An aggregated chronological group was used to provide a large enough sample size to analyse and assign players into the three-maturity status directed groups from. Consequently, the sample size for the players classified as ‘pre-PHV’ was lower compared to the ‘circa-PHV’ and ‘post-PHV’ groups. However, due to the biological status of the players within the study, any combination of age categories (e.g. U13 and U14) would have also provided one maturity status band of considerably smaller group number.

## Conclusions

Evidence here suggests that maturity status bio-banding likely creates homogeneous groups of players who are characterised by similar maturity-related anthropometric and physical fitness qualities. However, the further removed the measure becomes from biological maturation, the less effective maturity status bio-banding is on reducing between player, maturity-related differences. For instance, physical fitness does show some reduction in CV, but less than anthropometric measures. However, the effect of bio-banding on FMS™ characteristics is further reduced given the more tenuous link to biological maturation, despite maturation having an influence upon some measures of functional movement. It is suggested that equitable playing and training environments affords players with a beneficial development setting for young players and permitting the opportunity for them to express themselves technically and tactically without the pronounced challenge of more physically develop opposition [16, 17]. Therefore, it is proposed practitioners should consider using bio-banding to create homogenous (displaying similar anthropometrical and physical capacities) groups of players, which will create a more physically balanced playing and training environment and provide players with varied developmental stimulus. Subsequently offering an exclusive [21] solution to maturity-related selection bias of coaches [5, 21, 40]. Practitioners should consider bio-banding as one of the many tools at their disposal to assist their player retention decision making process and reduce the (sub)conscious selection of players based on transient maturity-related enhancements in anthropometric and physical characteristics which are often afforded to the earlier maturing players [14, 46–48]. Additionally, practitioners may wish to consider the format of bio-banded sessions. For instance, by maturity matching players who are early maturing and more anthropometrically and physically advanced may present these players with greater physical and technical challenges [15, 16]. Conversely, the use of maturity mis-matching players (players of differing maturity status competing) has been shown to elicit changes in psychological attributes which may facilitate the development of less developed players (e.g. ‘pre-PHV’), who when faced with the physical challenge presented by more physically advanced players, appear to increase their ‘competitiveness’ and ‘positive attitude’ [14, 49]. Lastly, findings here are limited to maturity-offset methods only and do not include measures of maturity derived from estimations of final parental height [50], which has been shown to possess superior prediction qualities that maturity offset methods [26].

In summary, practitioners should carefully consider their use and purpose of maturity status bio-banding. Maturity status bio-banding creates distinct groups which may assist in the improvement of talent identification by removing or increasing the physical challenge faced by players. Finally, players populating the ‘circa-PHV’ group should be carefully monitored for their duration within this phase on the account of decreased functional movement qualities, afflicting movement ability, and potentially resulting in increased risk of injury.
